# Electrothermal Vessel Sealing Versus Conventional Suturing in Abdominal Hysterectomy: A Randomised Trial

**DOI:** 10.7759/cureus.34123

**Published:** 2023-01-23

**Authors:** Pankhuri Dubey, Madhulika Dube, Anjali Kanhere, Neepa Biswas, Reena De, Arnab Koley, Pradip K Banerjee

**Affiliations:** 1 Obstetrics and Gynecology, All India Institute of Medical Sciences, Bhopal, IND; 2 Statistics, Babasaheb Bhimrao Ambedkar University, Lucknow, IND; 3 Obstetrics and Gynecology, Chirayu Medical College and Hospital, Bhopal, IND; 4 Obstetrics and Gynecology, Nilratan Sircar Medical College and Hospital, Kolkata, IND; 5 Obstetrics and Gynecology, Medical College, Kolkata, IND

**Keywords:** electrosurgery, ligasure, abdominal hysterectomy, hemostasis, electrothermal bipolar vessel sealers

## Abstract

Introduction: The present randomised controlled trial was conducted to compare haemostatic efficiency, operative time, and overall performance of the electrothermal bipolar vessel sealing (EBVS) system with conventional suturing in abdominal hysterectomy.

Materials and methods: The trial was designed with standard parallel arms, i.e., vessel sealing and suture ligature arms. Sixty patients were block randomised into either arms with 30 patients in each. A hand-held vessel sealing instrument was used to perform a hysterectomy in the vessel sealing arm and the quality of the uterine artery seal achieved at the first attempt was graded on an ordinal scale of 1-3 to quantify haemostatic efficiency. Operative time, intra-operative blood loss, and peri-operative complications were compared between the two arms.

Results: Significantly reduced mean operative time (26.97±8.92 vs 33.67±8.62 minutes; p=0.005) and intra-operative blood loss (111±53.31 mL vs 320±193.90 mL; p=0.001) was observed in the Vessel Sealing Arm compared to Suture Ligature Arm. Of total 60 uterine seals (from bilateral uterine artery transaction in 30 hysterectomies in the Vessel Sealing Arm), 83.34% were Level 1 with Complete Seal and no residual bleeding, 8.33% were Level 2 or Partial Seals with minimal bleeding, requiring the use of vessel sealers for a second time, while 8.33% had Seal Failure (Level 3) with significant bleeding requiring additional re-security of stumps with sutures. Modal pain scores on the first three postoperative days and duration of hospital stay were significantly less in the Vessel Sealer Arm, reflecting reduced postoperative morbidity. Outcomes were comparable across operators.

Conclusion: Vessel Sealing System gives superior surgical results with lesser operative time, minimal blood loss, and reduced morbidity.

## Introduction

Electrothermal bipolar vessel sealing (EBVS) devices have revolutionised both open and minimally invasive surgeries (MIS) by providing an effective alternative to sutures with their efficient and precise haemostatic sealing. Utilising an optimised combination of pressure and radiofrequency, vessel sealers generate targeted energy tailored to the tissue impedance and can efficiently seal blood vessels up to 7 mm in diameter with a thermal injury confined to 2 mm over the surgical site. With controlled energy delivery, a single instrument grasps, cuts, and creates distinct seals with burst strength comparable to clips and sutures [1], thus obliviating the need for multiple instruments during surgery. Numerous studies have shown their compelling applicability in a variety of open surgeries including hysterectomy through vaginal and minimally invasive routes [2-4]. However, data is conflicting for abdominal hysterectomy, as available studies have not shown any compelling applicability of the EBVS systems with respect to operative time and blood loss [5-7].

Abdominal hysterectomy remains the most common approach worldwide [8] and is the unquestioned stepping-stone towards understanding surgical anatomy and learning advanced gynaecological surgeries for young trainees. Even in the era of MIS, practical knowledge and competence in open surgeries with skill in performing hysterectomy via the abdominal route are indispensable, especially when surgery through the laparoscopic or vaginal route is not feasible or has to be abandoned on technical grounds. Learning and performing abdominal hysterectomy can be done with admirable ease by employing hand-held Vessel Sealers designed specifically for open surgery. As an efficient alternative to placing clamps and mechanical sutures in abdominal hysterectomy, the use of vessel sealers to directly grasp, cut and seal hysterectomy stumps can truly improve overall surgical performance with efficient haemostasis and shorter operative time. The present study compares the efficacy and overall surgical outcomes of vessel sealers versus conventional suturing in elective abdominal hysterectomy in a randomised controlled setting.

## Materials and methods

The study was conducted for a period of one year in the Department of Obstetrics and Gynaecology at a teaching hospital and tertiary care centre after approval from Institutional Review Board and Ethics Committee (Registration No. ECR/609/Inst/WB/2014). Standard parallel group randomised controlled trial design was employed to group participants into the intervention arms, i.e., abdominal hysterectomy using vessel sealers (Vessel Sealing Arm) or the conventional suturing technique (Suture Ligature Arm). LigaSure™ Max Hand Switching Reusable Instrument (Coviden, Valleylab LS10) was used for vessel sealing in our study. Choice of vessel sealer was based simply on its availability in our public sector hospital. No special funding was received for the study. A single instrument was re-sterilised and used up to eight times.

**Figure 1:**
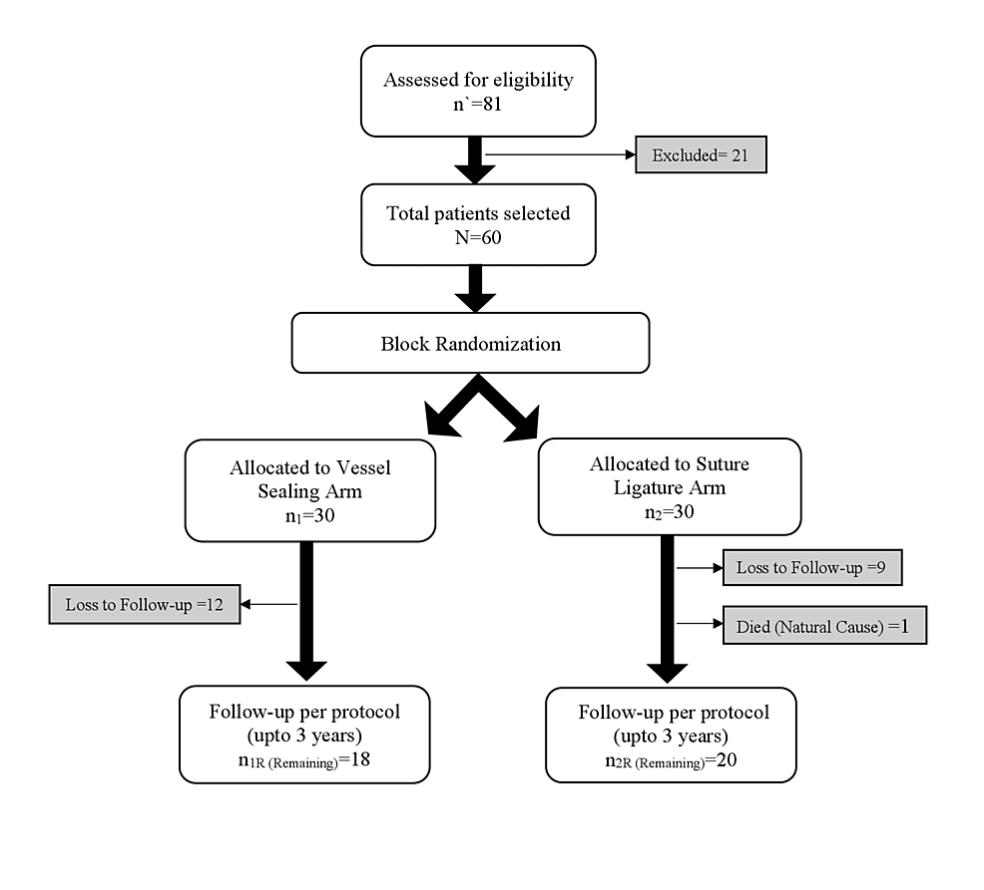
Flow chart illustrating parallel-group randomised controlled trial design of the study with two intervention arms, the Vessel Sealing Arm utilising EBVS system for abdominal hysterectomy, and the Suture Ligature Arm using conventional technique of clamping and suturing during hysterectomy

All patients posted for elective Abdominal Hysterectomy were assessed for eligibility; the criterion being benign disease. All cases with suspected or proven malignancy were excluded. Also, people with implanted electronic devices were not included as the vessel sealing technology may cause interference with the intended function of the former. After proper consent, 60 cases were selected using non-probability consecutive sampling and were then block randomised into Vessel Sealing and Suture Ligature arms to undergo hysterectomy with either vessel sealing or conventional technique, with 30 patients in each (Figure 1).

The sample size was estimated considering a 5% level of significance and a power of 80% with an assumed standard deviation of 20 minutes in mean operating time as described by a previous pilot study [5]. Case allocation to either the Vessel Sealing or Suture Ligature arm was done based on an allocation sequence generated using a block randomisation generator software with random permuted blocks of sizes four or six, for a total sample size of 60. Based on this allocation string, 60 opaque, sealed, and sequentially numbered envelopes were made in advance that contained case allocation instructions to either the Vessel Sealing or Suture Ligature arm.

The sealed envelopes were opened by the operating surgeon at the start of anaesthesia. The total participating surgeons were four: Senior consultants A & B, with more than ten years of surgical experience; and Junior Consultants C & D, with up to 5-10 years of experience. Operating surgeon was decided based on a pre-scheduled rota for the day and the envelopes did not influence who was to operate. The patients were blinded to surgical technique.

Amongst others, the following parameters were mainly noted: Patients’ BMI, history of previous pelvic surgery, indication for the current operation, duration of the operation (time taken from placement of the first clamp to vaginal vault closure with adequate haemostasis), blood loss (collection in suction cylinder bottle and soakage of dry lap sponges), use of intra-operative blood transfusions, grade or quality of the seal achieved using vessel sealers on uterine stumps, need of sutures for re-securing stumps in case of seal failure and postoperative complications.

The grade or quality of seal achieved on the uterine stump at the first attempt using vessel sealers in the Vessel Sealing Arm (First Attempt Seal) was classified intra-operatively on an ordinal scale of 1-3 defined in the following manner: Level 1, Complete or Satisfactory Seal with no bleeding; Level 2, Partial or Unsatisfactory Seal with minimal bleeding requiring reapplication of vessel sealers for a second time; Level 3, Seal Failure with Significant bleeding requiring stump security with additional sutures. Seal performance was deemed adequate if Level 1 or 2 was achieved at the first attempt.

Post-operative pain assessment was done using the Visual Analog Scale on days 1, 2, and 3 at a fixed time each day for a patient, with pain scores reported by patients as a number ranging from 1 to 10 (1=No pain; 10= Worst possible pain. Modal pain scores were compared, and a difference of 20% (as examined in an earlier pilot study) between the two treatment arms was considered clinically relevant [6]. An initial follow-up examination was done at six weeks and six months, and a further yearly telephonic follow-up was carried on for three years from the completion of the study.

Statistical analysis to compare outcomes between two groups was done using central trend measurements, Student's t-test, Chi-square, and Fisher's exact test. For assessing the effect of various factors on the duration of two surgical procedures, such as uterine size, history of previous pelvic surgery, BMI of patients, and analysis of Variance (ANOVA) was used. The level of significance was fixed at 0.05, and the p-value < 0.05 was deemed statistically significant. Data analysis was done using SPSS 21.

## Results

Preoperative variables

Patient characteristics presumed to potentially affect the outcome like age, BMI, size of the uterus, history of previous pelvic surgery, and indication for current surgery were well balanced between the study arms (Table 1).

**Table 1:** Baseline characteristics of patients randomized to the Vessel Sealing or the Suture Ligature Arm BMI: Body Mass Index; AUB-A: Abnormal Uterine Bleeding- Adenomyosis; AUB-L: Abnormal Uterine Bleeding-Leiomyoma; AUB-O: Abnormal Uterine Bleeding- Ovulatory Dysfunction; CIN 1: Cervical Intraepithelial Neoplasia 1 (low grade or mild dysplasia)

VARIABLE	VESSEL SEALING ARM (n=30)	SUTURE LIGATURE ARM (n=30)	P-VALUE
Mean age in years (Range)	43.9 ± 6.17 (32-57)	41.4 ± 4.917 (34-51)	0.08
BMI <25	23 (77%)	22 (73%)	0.77
BMI ≥25	7 (23%)	8 (27%)	0.76
Previous history of Pelvic Surgery	12 (40%)	14 (47%)	0.60
Uterine size <16 weeks	9 (30%)	7 (23%)	0.56
Uterine size ≥16 weeks	21 (70%)	23 (77%)	0.56
Indication for Hysterectomy			
AUB-A	4	7	0.99
AUB-L	15	8	
AUB-O	10	10	
Adnexal mass	0	1	
Chronic Pelvic Pain	0	2	
CIN 1	1	2	

Operative time

**Figure 2:**
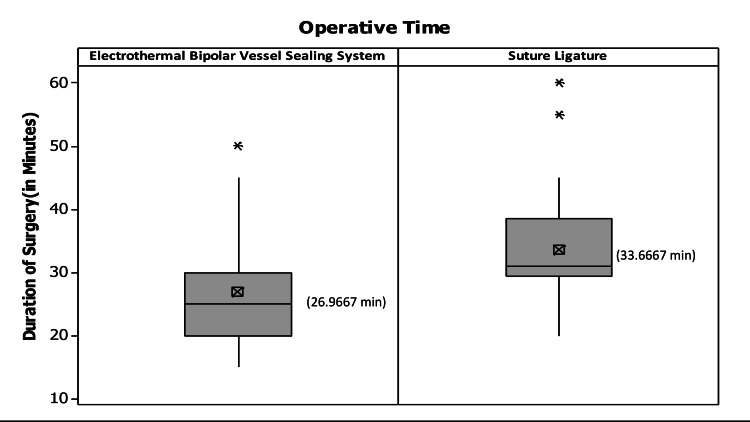
Box plots describing an operative time in vessel sealing arm versus suture ligature arm The central box spans the quartiles and shows the spread of the central half of the distribution of data on operative time. The horizontal line within the box marks the median and the smaller crossed ballot box represents the Mean for the data set. Lines (whiskers) extend out from either end of the box to extremes, representing the smallest and the largest noted values of the data set, respectively. Outliers are represented as data points outside the whiskers.

Operative time in each case was recorded as the time taken from placement of the first clamp to vaginal vault closure with adequate hemostasis. Mean operative time was 26.97±8.92 minutes using vessel sealers and 33.67±8.62 minutes with conventional suture ligature which was significant statistically (p=0.005). The full spread of data is pictorially represented using box plots in Figure 2.

The box plots describe skewed data spread of duration of surgery in the Conventional Suture Ligation technique while being symmetrical in Vessel Sealers, representing consistency of results with the Vessel Sealers in terms of performance. The difference in mean operative times were also not statistically significant for operating surgeons.

Additionally, factors assumed to cause a mechanical disadvantage during surgery, i.e. high BMI (≥25) of patients, large uterine size, and history of previous pelvic surgery with possible intra-peritoneal adhesions, which can theoretically increase the operative time, were statistically tested for their consequent effect on the duration of surgery by Analysis of Variance (ANOVA) using factorial analysis on Type of surgery (Vessel Sealer vs Sutures), uterine size (≥16 weeks vs <16 weeks), History of previous pelvic surgery (Yes or No), and BMI of patients (≥25 or <25). The idea of using factorial analysis was to assess the interaction effect of the aforementioned factors in addition to their individual effects.

Based on our data, the duration of surgery for the patients with higher BMI (≥25) operated using vessel Sealers was found to be significantly reduced (p = 0.001). Further, in the subgroup of these patients (BMI≥25) who had a history of previous pelvic surgery, and those with higher BMI and larger uterine size (≥16 weeks) were also found to have significantly shorter operative time with vessel sealers (p = 0.009 and p = 0.049 respectively) (Table 2).

**Table 2:** Factorial analysis on subgroup of patients with higher BMI, history of previous pelvic surgery, and larger uterine size to assess their interaction effect on duration of surgery in the two intervention groups

		VESSEL SEALER (Mean Time)	SUTURE LIGATURE (Mean Time)	P Value
DURATION OF SURGERY (in minutes)	Total	26.97 ±9.08	33.67± 8.77	<0.005
	Subgroup with BMI ≥ 25	30.33 ± 8.79	37.88 ± 10.30	<0.005
	Subgroup with BMI ≥ 25 and history of previous pelvic surgery	37.0 ± 4.0	39.50 ± 3.64	0.010
	Subgroup with BMI ≥ 25 and uterine size ≥ 16	30.42 ± 9.72	37.87 ± 10.30	0.049

Blood loss and uterine artery seal

**Figure 3:**
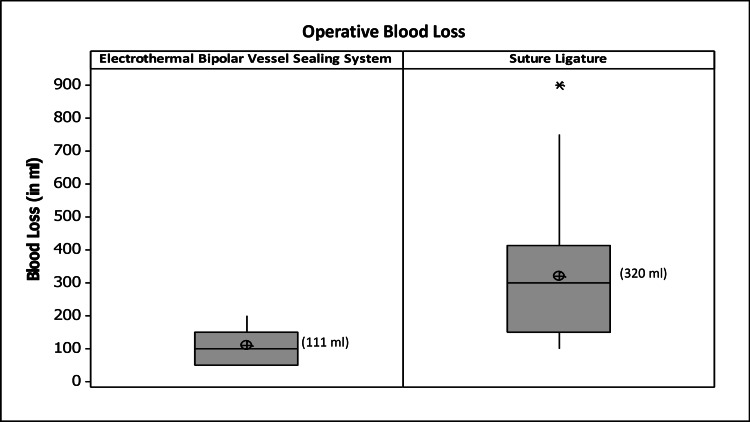
Box Plots describing blood loss in vessel sealing versus suture ligature arm The central box spans the quartiles and shows the spread of the central half of the distribution of data on operative time. The horizontal line within the box marks the median and the smaller crossed ballot box represents the Mean for the data set. Lines (whiskers) extend out from either end of the box to extremes, representing the smallest and the largest noted values of the data set, respectively. Outliers are represented as data points outside the whiskers.

 Average blood loss during hysterectomy with vessel sealers was 111±53.31 ml versus 320±193.90 ml with sutures, the difference being highly significant (p=0.001) (Figure 3).

Assessment of haemostatic efficacy of seal created on the uterine artery stump with EBVS in Vessel Sealing Arm was categorized into four grades, based on the quality of seal achieved at first attempt using vessel sealing device (Table 3).

**Table 3:** Categorisation of uterine seal achieved at first attempt with EBVS system

Quality of First Attempt Seal in Vessel Sealing Arm	n=60
Complete Seal (Level 1)	50 (83.4%)
Partial Sealing requiring reapplication (Level 2)	5 (8.33%)
Seal Failure; Stump re-secured with sutures (Level 3)	5 (8.33%)

Of the 60 uterine artery stumps (bilateral) that were grasped, cut, and sealed using vessel sealers in a total of 30 hysterectomies in the Vessel Sealing Arm, 83.34% of first attempt uterine artery seals were Level 1, with Complete and Satisfactory Seals requiring no additional intervention; 8.33% were Level 2, where Partial Seals were created at first attempt with minor bleeding, requiring a second attempt with vessel sealers to achieve a satisfactory seal; and 8.4% were Level 3 with Seal Failure and significant bleeding that required additional re-security of stumps with mechanical sutures (five out of 60 seals).

Of note, all Seal Failures were encountered either in large uteri of size 20 weeks and above (wherein uterine artery size was presumably more than 7 mm), or in patients with endometriosis with dense adhesions. No reactionary or secondary haemorrhage necessitating re-laparotomy was encountered in our study. The need for intra-operative blood transfusion was assessed by the anaesthesia team based on clinical metrics and maximum allowable blood loss. Transfusions were needed in two vs five cases (Vessel Sealing vs Suture Ligature).

Post-operative course

Pain Scores

Post-operative pain was assessed using the Visual Analogue Scale (1-10) on post-operative days 1, 2, and 3. Pain scores (Modal Values) were 6 vs 8 on postoperative day 1; 3 vs 6 on postoperative day 2; and 2 vs 3 on post-operative day 3. A significant difference of more than 20% was observed on all 3 days suggesting a more comfortable postoperative period in patients who underwent surgery with EBVS.

Duration of Hospital Stay

Mean duration of hospital stay (in days) was 5.43 in Suture Ligature arm versus 3.57 days in Vessel Sealing Arm including the day of surgery (p=0.000).

Return to Pre-operative Routine

Mean duration of return to pre-operative routine including routine self-care, light household activities etc. counting from the day of surgery was not significant between the two arms (7.5 days in Suture Ligature arm versus 6.50 days in Vessel Sealing Arm). However, women who were employed in light to moderate intensity paid physical work, i.e., 22 women in Suture Ligature Arm and 23 in Vessel Sealing Arm, returned to work in 15.77 days and 9.70 days, respectively, after surgery which was statistically significant (p=0.000).

## Discussion

Establishing efficient haemostasis is essential to provide a clear surgical field for adequate visualisation and improving overall clinical outcomes. Minimising blood loss during surgery becomes critical in peri-menopausal women with uterine pathologies, as the majority tend to be anaemic. The present study shows a clear advantage of the EBVS system over conventional suturing technique in terms of average blood loss, operative time, need for intra-operative blood transfusion, and post-operative recovery. Efficiency is further improved in obese patients, and in those with intra-peritoneal adhesions. Similar conclusions were drawn by Elhao et. al. in their trial on vaginal hysterectomy comparing vessel sealers versus sutures [9].

The Vessel Sealing System creates fused seal ends both laterally and medially, which prevents backflow of blood from the specimen side, thus enabling a virtually bloodless surgery. Adhesions restricting mobilisation of uterus wherever found were freed using Vessel Sealers which further decreased blood loss and hastened the surgery. Guo et al. in their network meta-analysis of haemostatic strategies for hysterectomy evaluated data from 20 studies comprising 1,392 patients and compared advanced bipolar vessel sealing system, conventional vessel sealing system, and Pituitrin based on the ease of use and haemostatic performance and concluded that Vessel Sealer application was best suited for reducing blood loss in specifically abdominal hysterectomy [10].

During abdominal hysterectomy, theoretically, the patient is subjected to more trauma as overall tissue handling is more when compared to other routes of hysterectomy. This is avoided in surgery with vessel sealers as direct tissue grasp along the length of the uterus, and simultaneous cutting and sealing of stumps using a single instrument subjects tissue bundles to minimal traction and crushing force, giving superior results [10].

The instrument used in our study was LigaSure™ Max Hand Switching Reusable Instrument that is 23 cm long, almost like a long curved haemostatic clamp, with jaw curved at a 30-degree angle containing 2.5 cm long electrode. It is easily held in either hand above the operative field to work in deeper areas of the pelvis to create seals up to 2.5 cm long and 3-5 mm wide. This was especially useful in obese patients with deep pelvis wherein passing a suture may be somewhat challenging, especially in the lower stumps. Also, the dissection of tissue bundles in patients with dense intra-peritoneal adhesions using the vessel sealer could be accomplished with greater finesse, as individual dissection and isolation of blood vessels within adhesion bands was not required, which minimised the chances of their avulsion and troublesome bleeding.

However, as with conventional technique, fenestration with adequate skeletonisation of uterine artery is imperative before application of the instrument in larger uteri with bulky parametrial tissue or when anatomy is distorted due to adhesions or fibroids. Doing so results in lesser seal failure and safeguards against any inadvertent ureteric injury. All 60 uterine artery seals (bilateral seals in 30 hysterectomies) were attempted after adequate skeletonisation and 91.7% of the seals were deemed adequate with no need of additional security with suture application (Level 1 and 2 Seals). Seal failure was encountered in five out of 60 seals, in a total of four cases, where uterine size was 20 weeks or greater. Of these, three cases had large multiple fibroids, and one had severe endometriosis with dense pelvic adhesions. The uterine artery diameter in these cases was seemingly more than 7 mm, which presumably led to seal failure. Nevertheless, with an overall lesser instrument exchange and efficient haemostasis, a statistically significant tendency towards shorter operative time was noted with vessel sealers when compared with the conventional suture ligature technique.

While studies on vaginal hysterectomy have drawn similar conclusions, interestingly, few previous trials on abdominal hysterectomy have not reported significant advantage with vessel sealers in regard to operative duration, statistically or clinically [5,6]. One reason for this difference could be the inclusion of time taken for peritoneal entry after incision for abdominal hysterectomy in their analysis which could introduce bias if certain patient characteristics (e.g., BMI) are not well balanced in the intervention groups. This was circumvented in our study by comparing the time taken for surgery, starting from placement of the first clamp to the closure of vaginal vault with haemostasis achieved to primary surgeon’s satisfaction.

Satisfactory dissection and advancing deep into the pelvis with ease in restricted pelvic space becomes extremely valuable in oncosurgery, where extensive pelvic dissection on fragile neoplastic tissue may be achieved with minimal tissue handling. Tedious operation steps are effectively avoided, the time of surgical instrument replacement is reduced with an overall reduction in operative duration as compared with conventional method [11]. A positive association is known between the operative duration and complications such as anaesthesia related morbidity, surgical site infection, venous thromboembolism, bleeding and haematoma formation, necrosis, etc., across various surgical procedures [12,13]. Duration of surgery remains an independent and potentially modifiable risk factor for these complications and its reasonable reduction should be a pragmatic intent for care providers [14]. Though a statistically significant difference of approximately seven minutes, as noted in the present study, may not seem significant clinically for routine hysterectomy, the proportionate reduction in surgical time and associated complications may prove truly invaluable in pelvic oncosurgery where average operative durations are inherently longer.

There is a generalised decrease in the amount of bleeding, operative mechanical trauma, peri-operative organ or tissue injury, and postoperative risk of deep venous thrombosis of the lower extremity. Absence of transfixing sutures prevent development of foreign body reaction, local ischaemia and necrosis, thus reducing the attendant post-operative pain and morbidity [15]. This translates to quicker return to work, and therefore reduced net income loss, as evidenced in the present study. 

We employed the modified Richardson technique of intrafascial hysterectomy in both treatment arms. In the technique, after the last pair of clamps are placed across the vagina below cervix, vagina is divided with knife, the uterus with cervix is delivered outside the operative field, and the vaginal apex is closed with sutures, keeping clamps in place. This is the closed cuff technique where the vagina is never exposed, and pelvic contamination is prevented. This was, however, not possible with all cases in the vessel sealing arm as the corresponding step involved grasping together the anterior and posterior vaginal walls with the jaws of the vessel sealer instrument and cutting across one half of the vaginal apex at a time. The resultant vaginal cuff opens into the unsterile vaginal canal and there is a risk, howsoever little, of pelvic contamination with vaginal secretions. An important consideration at this step, highlighted by Gizzo et al., is the possibility of vault prolapse due to thermal damage to the uterosacral ligament by the LigaSure system [4]. Although their study was on vaginal hysterectomy, the same can be theoretically expected in abdominal hysterectomy. We accomplished division of vagina while incorporating uterosacral ligaments in a single step without any immediate peri-operative suggestion of injury. Further, on follow-up till three years after the surgery, no vault descent/prolapse was reported in patients who underwent surgery with vessel sealers. Thus, no thermal injury to uterosacral ligaments or other collateral tissues was apparent in our study. 

The major disadvantage with system is the expense incurred. As per current list price, initial investment in a single, re-usable instrument with disposable electrode is $1,440, and each fresh pair of blades for LigaSureTM Max Hand Switching Reusable Instrument is listed at approximately $180 per pair. We could perform up to eight procedures with a single electrode, thus the instrument cost per procedure in our study came out to be around $64. This represents a direct addition to the cost of the procedure, which is significant especially in hospital settings catering to mostly indigent population within limited funds. However, days gained through quicker recovery as evidenced by faster return to work in the vessel sealing group may justify the higher cost.

## Conclusions

The present study shows that cost notwithstanding, the EBVS system has a clear advantage over conventional suturing techniques in terms of average blood loss, operative time, need for intra-operative blood transfusion, and post-operative recovery. With the delivery of consistent and precise haemostatic seals, the EBVS system promises superior surgical results, faster surgical recovery, and reduced morbidity.

Even with limited operator experience, the system reduces operative time and blood loss by a significant margin. A single EBVS instrument serving multiple purposes improves the accessibility of the surgical plane in a limited space and demonstrates further relevance in cases where mechanical disadvantage is apparent. Further studies are needed to evaluate its role in oncosurgery where the system may prove indispensable in achieving superior surgical results with minimal peri-operative morbidity.
